# Using texture and colour enhancement imaging to evaluate gastrointestinal diseases in clinical practice: a review

**DOI:** 10.1080/07853890.2022.2147992

**Published:** 2022-11-24

**Authors:** Mitsushige Sugimoto, Yohei Koyama, Takao Itoi, Takashi Kawai

**Affiliations:** a Department of Gastroenterological Endoscopy, Tokyo Medical University Hospital, Tokyo, Japan; b Department of Gastroenterology, Tokyo Medical University Hospital, Tokyo, Japan

**Keywords:** Texture and colour enhancement imaging, image-enhanced endoscopy, narrow-band imaging, ultrathin endoscopy, white-light imaging

## Abstract

White light imaging (WLI) is the most common endoscopic technique used for screening of gastrointestinal diseases. However, despite the advent of a new processor that offers sufficient clear illumination and other advanced developments in endoscopic instrumentation, WLI alone is inadequate for detecting all gastrointestinal diseases with abnormalities in mucosal discoloration and morphological changes to the mucosal surface. The recent development of image-enhanced endoscopy (IEE) has dramatically improved the detection of gastrointestinal diseases. Texture and colour enhancement imaging (TXI) is a new type of IEE that enhances brightness, surface irregularities, such as elevations or depressions, and subtle colour changes. TXI with two modes, namely modes 1 and 2, can selectively enhance brightness in dark areas of an endoscopic image and subtle tissue differences such as slight morphological or colour changes while simultaneously preventing over-enhancement. Several clinical studies have investigated the efficacy of TXI for detecting and visualizing gastrointestinal diseases, including oesophageal squamous cell carcinoma (ESCC), Barret’s epithelium, gastric cancer, gastric mucosal atrophy and intestinal metaplasia. Although TXI is often more useful for detecting and visualizing gastrointestinal diseases than WLI, it remains unclear whether TXI outperforms other IEEs, such as narrow-band imaging (NBI), in similar functions, and whether the performance of TXI modes 1 and 2 are comparable. Therefore, large-scale prospective studies are needed to compare the efficacy of TXI to WLI and other IEEs for endoscopic evaluation of patients undergoing screening endoscopy. Here, we review the characteristics and efficacy of TXI for the detection and visualization of gastrointestinal diseases.

**Key Messages**
- TXI mode 1 can improve the visibility of gastrointestinal diseases and qualitative diagnosis, especially for diseases associated with colour changes.
- The enhancement of texture and brightness with TXI mode 2 enables the detection of diseases, and is ideal for use in the first screening of gastrointestinal tract.

## 1. Introduction

Early detection through screening programmes that involve performing upper gastrointestinal endoscopy at health check-ups is widely known to reduce gastric cancer mortality. The strategy is particularly effective in East Asian countries, where the prevalence of *Helicobacter pylori* strains with potent virulent factors and gastric cancer are high [1]. However, endoscopic detection of oesophageal cancer, gastric cancer and pre-cancerous lesions, such as gastric atrophy, intestinal metaplasia and Barrett’s epithelium, using white-light imaging (WLI) alone has remained difficult and highly dependent on the expertise of the endoscopist. Thankfully, recent advances in image-enhanced endoscopy (IEE), including narrow-band imaging (NBI), blue laser imaging (BLI) and linked colour imaging (LCI), have improved the detection rate of gastrointestinal diseases, such as gastric cancer and intestinal metaplasia [2–8], oesophageal adenocarcinoma (EAC) [9,10] and Barrett’s epithelium [11,12]. Therefore, the advent of IEE is considered one of the most revolutionary developments in endoscopic equipment in recent years. In fact, the combination of WLI and IEE is recommended when performing endoscopic screening at health check-ups and in daily clinical practice.

Texture and colour enhancement imaging (TXI) was launched as a new optical IEE modality in April 2020. TXI, which utilizes Retinex theory-based image processing technology, enhances three imaging factors in WLI (texture, brightness and colour) and facilitates the clear definition of subtle tissue differences (e.g. normal mucosa, atrophy, dysplasia, and cancer) [13–15]. TXI is often more useful than WLI for detecting and visualizing gastrointestinal diseases (e.g. pharyngeal and oesophageal squamous cell carcinoma [PSCC and ESCC]) [14], gastric cancer [15,16], gastric atrophy [7,15], and intestinal metaplasia [7] as it selectively enhances brightness in dark areas of an endoscopic image and subtle tissue differences. However, it is unclear whether TXI outperforms other IEEs including NBI and LCI [13–18] in terms of detection and visualization. At present, the data on endoscopic screening using TXI in clinical practice remain preliminary as only a small number of cases are available for analysis [7,14–16,19–23].

Endoscopic examination in Japan is often performed transnasally using an ultrathin endoscope to reduce invasiveness and distress to the patient, especially in annual health check-ups and private clinic settings [24,25]. Although previous first- and second-generations of this technology had major disadvantages, including the need for complex planning, poor image quality, and a lower disease detection rate [26], third-generation high-vision ultrathin endoscopic technology, namely GIF-1200N (Olympus Co., Tokyo, Japan), provides markedly improved image quality and colour differences between normal gastric mucosa and intestinal metaplasia [27]. High-vision ultrathin endoscopy using TXI was recently shown to be even more useful for detecting and visualizing gastrointestinal diseases.

In this review, we provide an overview of the characteristics and efficacy of TXI for detecting and visualizing gastrointestinal diseases.

## 2. Characteristics of texture and colour enhancement imaging

**Figure 1.**
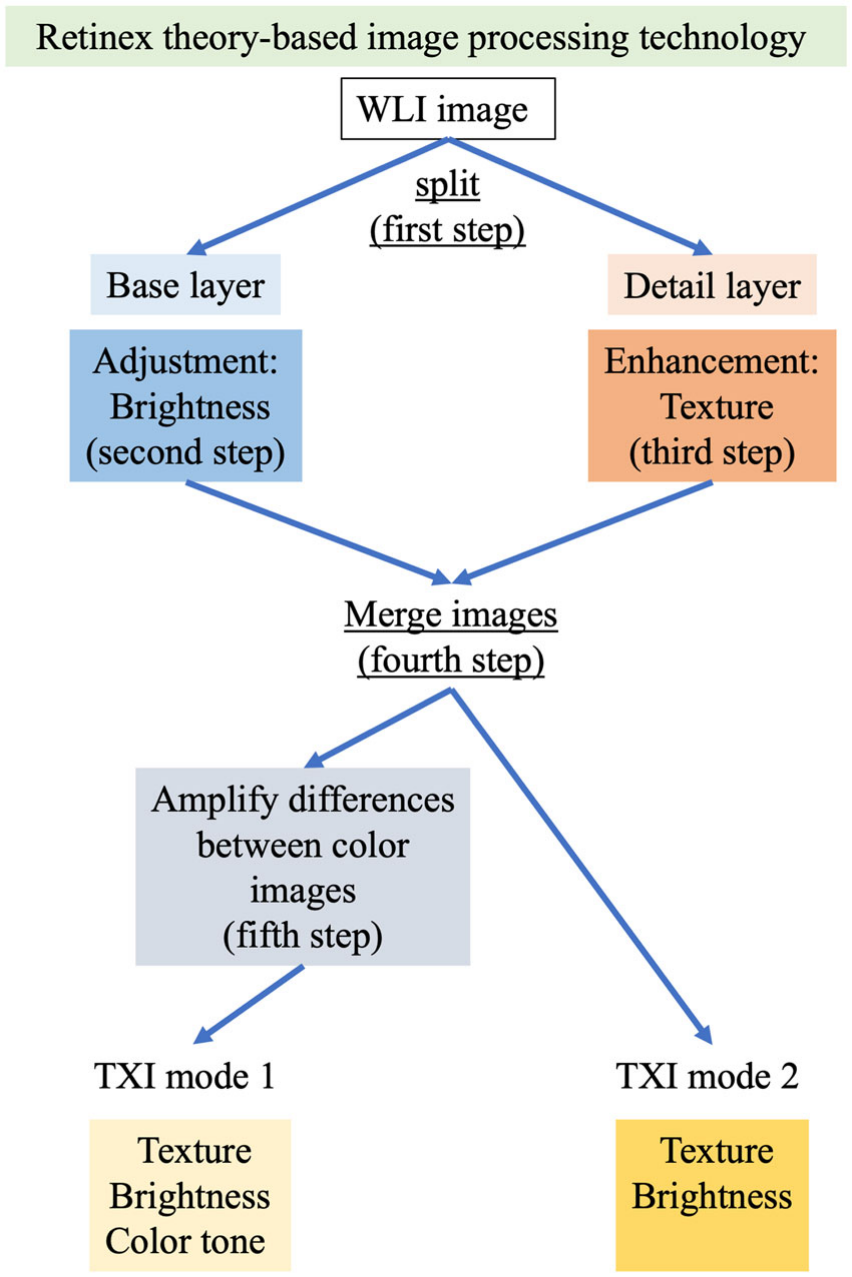
Scheme for the Retinex theory-based image (texture and colour enhancement imaging, TXI) processing technology [13]. The first step is to separate the image into a detail layer and a base layer. The second step is to adjust the brightness of the base layer in the white-light imaging (WLI) image such as to selectively brighten dark areas in the endoscopic image. The third step is to enhance the detail layer using processing techniques to highlight textures and improve contrast. After adjusting the brightness and enhancing textures, the fourth step is to merge the two layers to obtain TXI mode 2. The fifth step is to adjust the colour tone to obtain TXI mode 1.

TXI enhances three main imaging factors, namely texture, brightness and colour. The TXI system is needed to use a new processor called EVIS X-1, which produces improved image quality over older processors, such as EXERA III and LUCERA ELITE (Olympus Corporation, Tokyo, Japan) (Figure 1). This technology was developed with the aim of detecting all gastrointestinal diseases with abnormalities in mucosal discoloration and morphological changes to the mucosal surface by enhancing the texture, brightness and colour of endoscopic images.

According to the TXI algorithm, the first step of Retinex theory-based image processing is to separate the WLI image into a detail layer (texture) and a base layer (brightness) (Figure 1) [28]. The second step is to adjust the brightness of the base layer in the WLI image, such as to selectively brighten dark areas in the endoscopic image. The third step is to enhance the detail layer using processing techniques to highlight textures and improve contrast. Adjustment of brightness is a challenging step that is often limited by the reflection of light originating from brighter areas of the image. After adjusting the brightness and enhancing textures, in the fourth step, the two layers are merged and outputted as the endoscopic image for TXI mode 2. In the fifth step, colour tones are augmented to amplify differences in colour images, particularly between the bandwidths of white and red. This image is outputted as the endoscopic image for TXI mode 1. Therefore, the image in TXI mode 2 has enhanced imaging factors of texture and brightness and the image in TXI mode 1 has enhanced imaging factors of texture, brightness and colour tone. The endoscopic image in TXI mode 2 is closer in colour tone to the WLI image compared with that in TXI mode 1.

Of IEEs, NBI is an optical technology that modifies the centre wavelength and bandwidth of an endoscope’s light into a narrow-band illumination (two wavelengths, 415 and 540 nm). By utilizing this narrow spectrum, the contrast in the capillary pattern of the superficial layer is markedly improved [29], thereby facilitating clearer visualization of vascular structures [30]. LCI emits short wavelengths of 410 and 450 nm in addition to the wavelength of WLI, and produces particularly bright images [31]. The advantages of LCI for detecting tumours are based on short-wavelength reflection in various degrees from the superficial mucosal layer and colour difference expansion [32]. The blue laser endoscopy system adopts two different wavelengths of lasers: white light laser (450 nm) and BLI laser (410 nm). BLI laser, with a shorter wavelength and narrow spectrum, presents a narrow-band light image, which can highlight the capillaries and microstructure of the mucosal surface, so as to observe the pathological changes in microscopic structure [33]. However, TXI is the post-processing technology, which is the emphasis (texture, brightness and colour) by image processing, and TXI is not processed to emphasize the specific wavelength of light, as observed in other IEEs including NBI, BLI and LCI [18].

## 3. Usefulness of TXI for detecting gastrointestinal diseases

### 3.1. Pharyngeal and oesophageal squamous cell carcinoma

The International Agency for Research on Cancer estimates that 450,000 and 572,000 patients had oesophageal cancer worldwide in 2012 and 2018, respectively, and the number of patients with oesophageal cancer is increasing every year. Of those with oesophageal cancer, 84% had ESCC and 15% had EAC related to Barret’s oesophagus and reflux esophagitis in 2018 [34,35]. The geographic distribution of ESCC varies worldwide, and the prevalence of ESCC in Asian countries is more than 80% of that recorded globally [35]. Major risk factors for ESCC include smoking, alcohol consumption, alcohol by volume, genetic factors, such as variations in the genes encoding aldehyde dehydrogenase-2 and the low-activity form of alcohol dehydrogenase 1B, low consumption of fruits and vegetables, diet, consumption of hot foods and beverages, body mass index, and socioeconomic status [36–40]. Although ESCC occasionally develops synchronously or metachronously in the oesophagus as well as the pharynx, larynx and oral cavity, the prognosis of ESCC is generally poor when it is detected at an advanced stage [41]. Therefore, careful annual endoscopic examination in patients with major risk factors is useful for early detection of ESCC.

Recognition of colour and morphological changes, and identification of tumour vessels is essential for early ESCC detection during an endoscopic procedure [42]. WLI does not endoscopically detect ESCC or EAC in all patients [43,44]. The sensitivity of WLI for detecting superficial ESCC and PSCC is 55% and 8%, respectively, even among expert endoscopists using high-definition endoscopic instruments [43]. A randomized-controlled trial (RCT) that compared WLI and NBI for endoscopic detection of ESCC in high-risk patients reported that NBI detects SCC more frequently than WLI (head/neck: 100 *vs*. 8% and ESCC: 97 *vs.* 55%, respectively) [43]. Therefore, NBI is currently the standard technique for detecting PSCC and ESCC.

**Figure 2.**
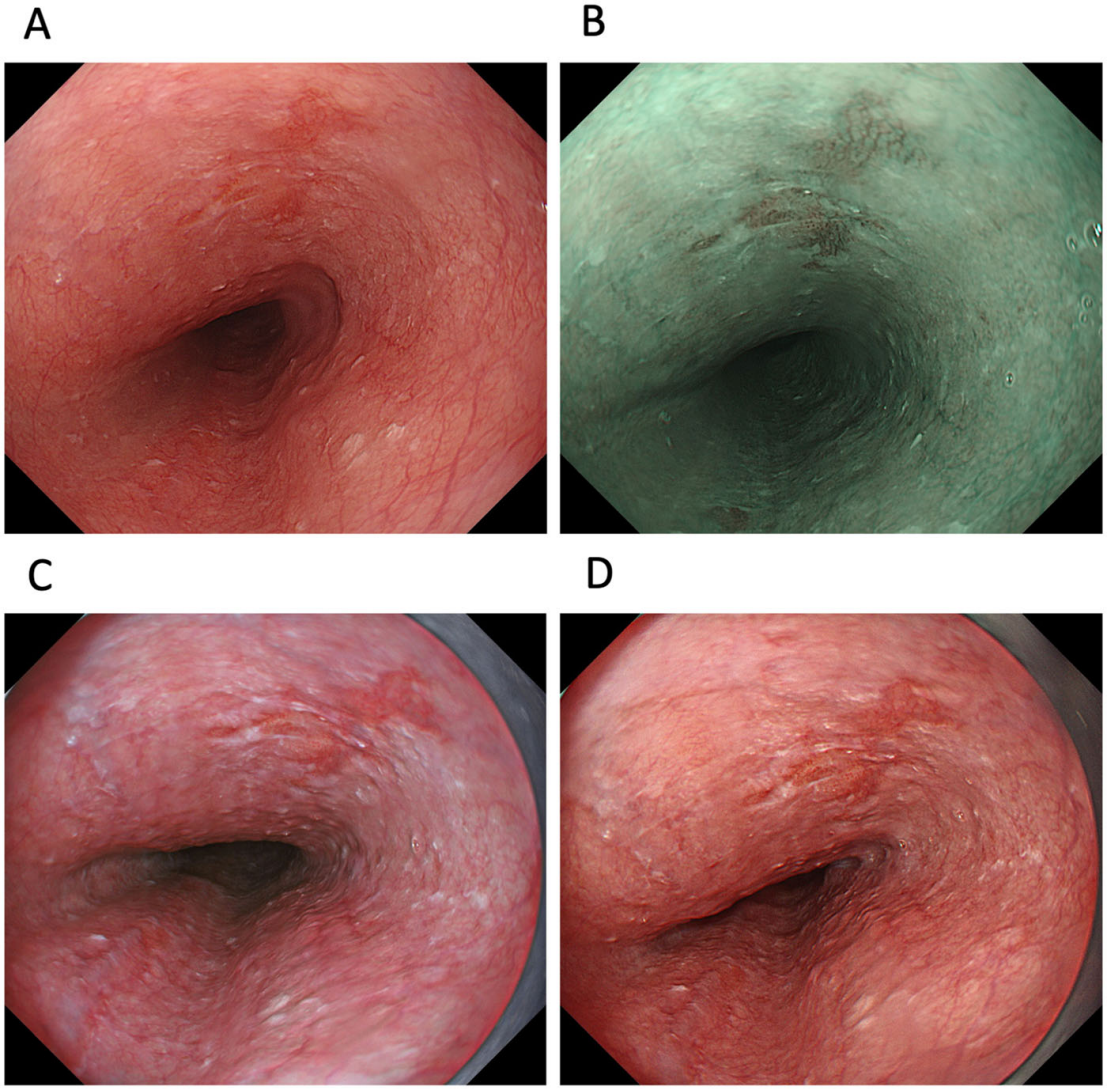
A case of oesophageal squamous cell carcinoma (ESCC). ESCC observed by (A) white-light imaging (WLI), (B) narrow-band imaging, (C) texture and colour enhancement imaging (TXI) mode 1 and (D) TXI mode 2. Compared to WLI, TXI modes 1 and 2 emphasize the redness of the ESCC, making the boundary with the surrounding non-carcinoma normal oesophageal mucosa clear.

One study examined the usefulness of TXI for detecting PSCC and ESCC (Tables 1 and 2, and Figure 2). Colour differences are an important factor in the detection of PSCC and ESCC. The study showed that the mean colour difference based on the CIE 1976 (L*, a*, b*) colour space [45,46] between ESCC and the surrounding oesophageal mucosa was 12.6 ± 5.4 in WLI, 19.8 ± 5.6 in TXI mode 1, 15.2 ± 4.9 in TXI mode 2 and 19.2 ± 7.1 in NBI, with the colour difference of TXI mode 1, mode 2 and NBI being significantly higher than that of WLI (*p* < 0.001) [score 1 (improved visibility of lesion), 0 (unchanged visibility of lesion) and −1 (worsened visibility of lesion)] [14]. The endoscopic visibility of ESCC improved by 62.5% in TXI mode 1 and by 88.1% in NBI compared with images in WLI. This enhanced visibility was also observed for low-grade intraepithelial neoplasia, high-grade intraepithelial neoplasia and PSCC [14]. However, the authors noted no significant correlation between the visibility score and tumour factors, including location (pharynx *vs.* oesophagus), macroscopic type (IIb *vs.* IIa or IIc) and tumour size (<10 mm *vs.* >10 mm) [14]. Although TXI mode 1 enhances colour changes and improves visibility through colour enhancement of PSCC and ESCC compared to WLI and TXI mode 2, TXI mode 1 may be more useful for detecting whitish- or reddish-coloured PSCC and ESCC from the surrounding pharyngeal and oesophageal mucosa. Therefore, enhancing the colour tone may improve the visibility of PSCC and ESCC, but may not be sufficient to emphasize structure [14]. However, evidence concerning the efficacy of TXI for the detection of ESCC is preliminary. When TXI can provide data overcoming NBI in the detection of PSCC and ESCC, TXI will be valuable endoscopic technology. However, at present, it is unclear whether TXI should be the standard examination technique in the detection of PSCC and ESCC. Because NBI is currently the standard technique for detecting PSCC and ESCC, further studies are needed to evaluate the various IEE modalities including NBI and TXI for detecting PSCC and ESCC.

**Table 1.** Colour differences in gastrointestinal diseases detected using various image-enhanced endoscopy techniques.

Disease		Number of patients/ lesions	Factor	WLI	TXI mode 1	TXI mode 2	NBI	*p* Value (WLI *vs.* TXI mode 1)	*p* Value (WLI *vs.* TXI mode 2)	*p* Value (NBI *vs.* TXI mode 1)	*p* Value (TXI mode 1 *vs*. 2)
Oesophagus											
SCC	Dobashi et al. [14]	30/59		11.6. ± 4.8	18.6 ± 6.8	14.3 ± 5.5	17.2 ± 7.3	<0.001	<0.001	NA	NA
		/24	SCC	12.6 ± 5.4	19.8 ± 5.6	15.2 ± 4.9	19.2 ± 7.1	<0.001	<0.001	NA	NA
		/11	HGIN	11.4 ± 3.1	19.6 ± 7.2	14.7 ± 2.7	15.4 ± 4.5	0.001	0.001	NA	NA
		/16	LGIN	10.0 ± 3.4	16.0 ± 5.7	12.1 ± 4.4	14.7 ± 5.4	0.001	0.001	NA	NA
		/15	pharynx	10.4 ± 3.8	16.2 ± 5.6	13.3 ± 4.8	13.8 ± 5.9	<0.001	<0.001	NA	NA
		/44	oesophagus	11.9 ± 5.1	19.4 ± 7.0	14.7 ± 5.8	18.4 ± 7.4	<0.001	<0.001	NA	NA
Stomach											
Atrophy	Ishikawa et al. [15]	19/		8.7 ± 4.2	14.2 ± 8.0	10.0 ± 4.2		0.009	0.017	NA	0.261
	Sugimoto et al. [7]	30/		14.5 ± 5.9			19.3 ± 8.0	NA	NA	NA	NA
		30/		14.0 ± 7.3		20.8 ± 9.7		NA	<0.001	NA	NA
Intestinal metaplasia	Sugimoto et al. [7]	30/		6.6 ± 3.2			13.5 ± 4.7	NA	NA	NA	NA
		30/		6.5 ± 3.1		10.9 ± 3.8		NA	0.003	NA	NA
Map-like redness	Sugimoto et al. [7]	30/		5.8 ± 3.7			11.7 ± 9.3	NA	NA	NA	NA
		30/		13.1 ± 3.1		13.5 ± 5.6		NA	0.885	NA	NA
Gastric cancer	Ishikawa et al. [15]	11/12		8.0 ± 4.2	18.7 ± 16.0	10.2 ± 8.4		<0.01	0.831	NA	0.042
			Indigo carmine spraying	11.2 ± 8.0	28.1 ± 12.7	16.8 ± 11.2		<0.01	0.151	NA	<0.01
	Abe et al. [16]	18/20		10.3 ± 4.7	15.5 ± 7.8	12.7 ± 6.1		0.04	0.61	NA	0.54
Colon											
Non-polypoid colorectal lesions (< 20 mm)	Yoshida et al. [19]	26/101		9.7 ± 6.0	13.3 ± 6.3		13.1 ± 6.8	<0.001	NA	0.81	NA
		/43	SSL + HP	7.0 ± 2.8	11.1 ± 4.4		12.6 ± 6.0	<0.001	NA	0.07	NA
		/58	LGD + HGD + T1 cancer	11.8 ± 7.0	14.9 ± 7.1		13.5 ± 7.4	<0.001	NA	0.09	NA

HGIN: high-grade intraepithelial neoplasm; HGD: high-grade dysplasia; HP: hyperplastic polyp; LGIN: low-grade intraepithelial neoplasm; LGD: low-grade dysplasia; NA: not available; NBI: narrow-band imaging; SCC: squamous cell carcinoma; SSL: sessile serrated lesions; TXI: texture and colour enhancement imaging; WLI: white-light imaging

### 3.2. Barret’s oesophagus and reflux esophagitis

While the incidence of ESCC has been dramatically decreasing in Western countries, the incidence of EAC has increased. This is thought to be due to the decrease in *H. pylori* infection and increase in reflux esophagitis and Barret’s oesophagus [35,47,48]. Although Barrett’s oesophagus is clinically recognized as requiring routine and careful endoscopic surveillance [49,50], long-segment Barrett’s oesophagus is an especially well-known major risk factor for EAC. The European Society of Gastrointestinal Endoscopy recommends endoscopic surveillance of Barrett’s oesophagus using high-definition WLI, followed by a random biopsy of Barrett’s epithelium in the absence of any lesions [51]. However, this random biopsy has drawbacks: the area of biopsied tissue sampled accounts for less than 2–5% of the total area of Barrett’s epithelium [52]. To overcome this disadvantage, there has been a recent increase in the use of NBI and BLI for surveillance endoscopy to evaluate the presence Barrett’s epithelium, dysplasia and EAC [2–6]. In addition, a recent meta-analysis reported that the sensitivity, negative predictive value, and specificity of endoscopic surveillance for Barrett’s oesophagus using NBI was 94.2% (95% CI, 82.6–98.2%), 97.5% (95.1–98.7%) and 94.4% (80.5–98.6%), respectively [53]. Compared with WLI-targeted biopsy for Barrett’s oesophagus, NBI-targeted biopsy shows higher diagnostic accuracy for detecting dysplasia, with a sensitivity of 76%, specificity of 99%, positive predictive value of 97% and negative predictive value of 84% [54]. Therefore, surveillance endoscopy using NBI is considered an effective method for evaluating Barrett’s oesophagus related with EAC worldwide [2–6,53,54].

**Figure 3.**
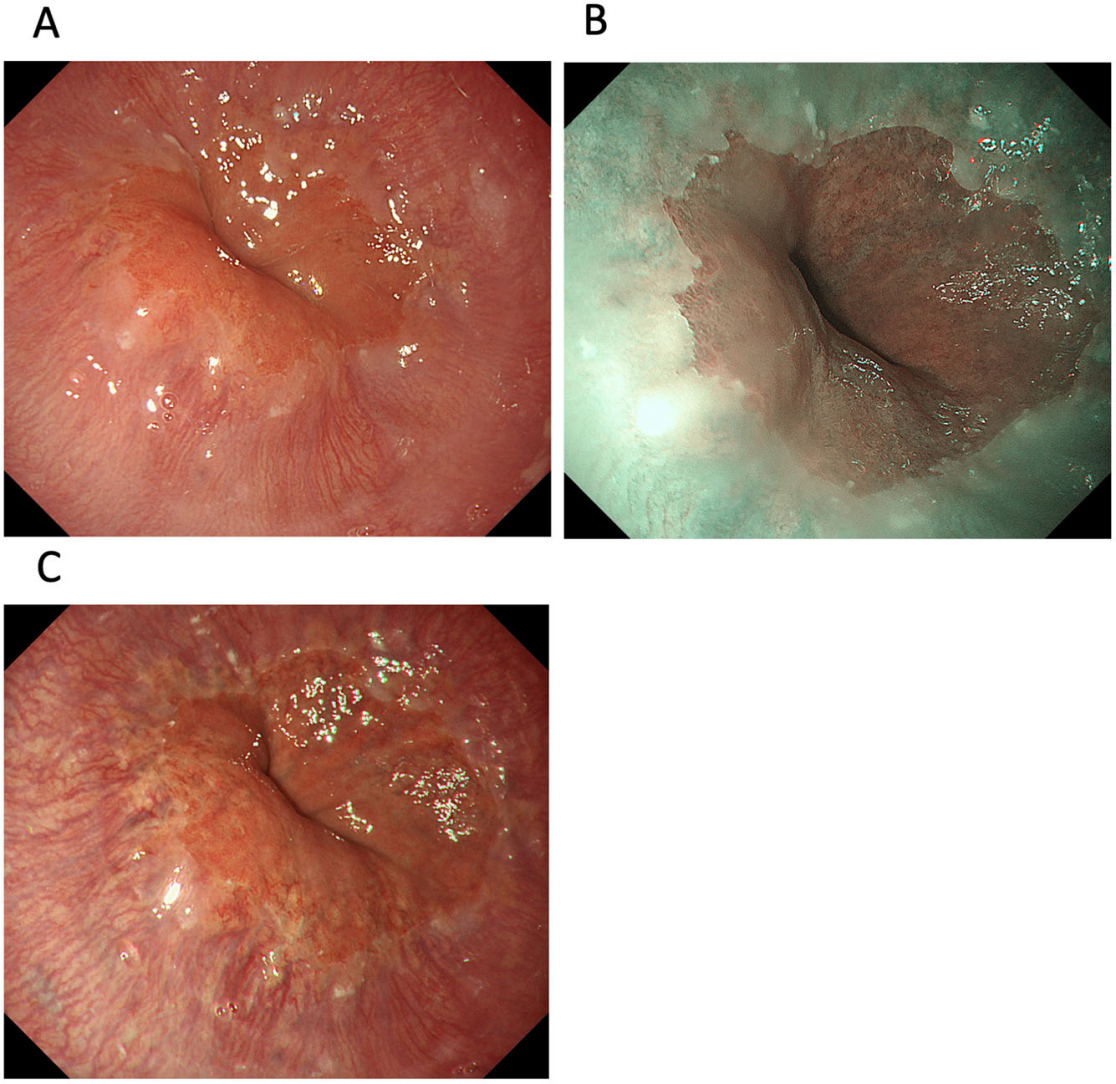
Images of Barrett’s epithelium taken using a third-generation ultrathin endoscope with white-light imaging (WLI) (A), narrow-band imaging (B), and texture and colour enhancement imaging (TXI) mode 2 (C). TXI mode 2 shows whitish changes in the oesophagocardial junction more clearly than WLI.

In terms of Barrett’s epithelium and reflux esophagitis, there is no published report investigating detection and endoscopic grading among WLI, NBI and TXI mode 2. As our preliminary data, high-vision ultrathin endoscopy allows TXI mode 2 and NBI to reveal significantly greater colour differences to differentiate Barrett’s epithelium from the gastric mucosa than WLI (Figure 3). Interestingly, gastroesophageal reflux disease (GERD) grade M (whitish turbidity in the oesophagogastric junction) is more clearly visible on TXI mode 2 than WLI. Therefore, because evaluation using both NBI and TXI mode 2 may be useful for identifying patients with Barrett’s oesophagus who are at high risk of EAC at health check-ups, prospective studies examined the efficacy of TXI mode 1 for evaluating Barrett’s epithelium, reflux esophagitis, or EAC, are needed to do so in the future.

### 3.3. Gastric atrophy and intestinal metaplasia

Gastric cancer is generally caused by a multifactorial and multistep process, and severe atrophy and intestinal metaplasia related to long-term *H. pylori* infection are major risk factors for gastric cancer [55–57]. Further, the map-like redness that represents histologic intestinal metaplasia is a new endoscopic risk factor in patients who have received *H. pylori* eradication therapy [3,58]. In Japan, the Kyoto classification of gastritis provides a system for grading endoscopic risk factors for gastric cancer [56,59,60]. Based on this system, patients are divided into three groups: those who are *H. pylori*-negative, those with current infection, and those who have previously been infected. They are then assessed for gastric cancer risk by scoring five endoscopic parameters, namely atrophy, intestinal metaplasia, enlarged folds, nodularity and diffuse redness [56,59,60]. Kyoto gastritis scores for atrophy and intestinal metaplasia are significantly higher in gastric cancer patients than in subjects with gastritis alone. Further, multivariate analysis has demonstrated that the risk of gastric cancer in elderly patients increases with atrophy (OR: 1.822, 95% CI: 1.087–3.056) and intestinal metaplasia (5.954, 4.157–8.527) [56]. Moreover, the MAPS II guideline states that patients with intestinal metaplasia and severe atrophy (OLGA/OLGIM Stages III and IV) should be followed up with a high-quality endoscopy every 3 years [61,62]. Therefore, correct evaluation of gastric atrophy, intestinal metaplasia and map-like redness in high-risk patients is important for reducing gastric cancer mortality.

IEE improves the visibility of endoscopic findings and accuracy of endoscopic diagnosis of intestinal metaplasia [4,63]. Characteristics of intestinal metaplasia that can be observed by NBI on magnifying endoscopy are the white opaque substance on the gastric epithelium and the light blue crest on the mucosal epithelial rim [64]. In addition, typical greyish-white elevations, undetected lesions and patchy redness in WLI appear lavender in LCI [4]. Therefore, the European guideline states that using a high-definition endoscope with IEE that includes NBI and LCI is more effective for detecting atrophy and intestinal metaplasia than high-definition WLI alone [61].

**Figure 4.**
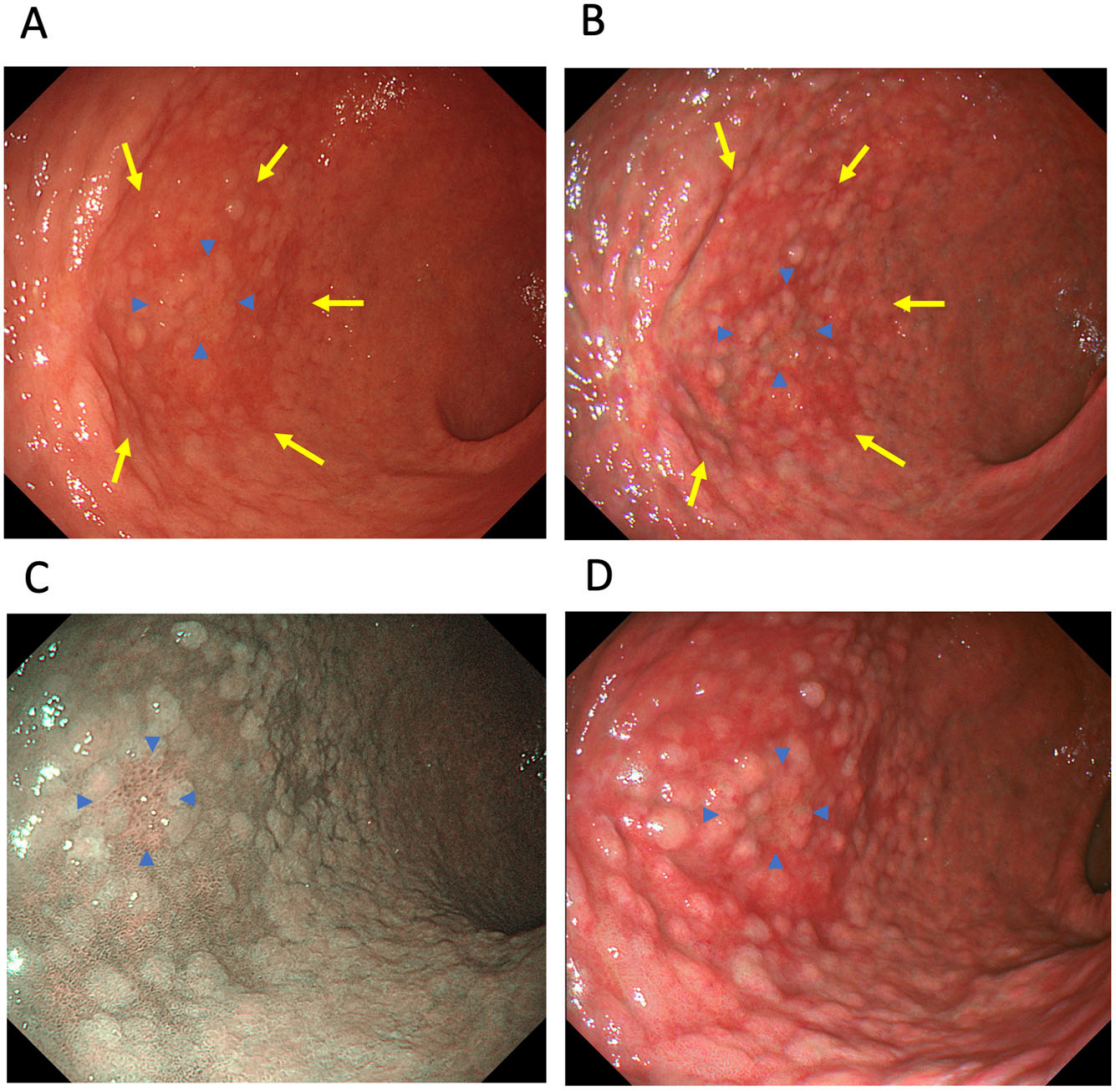
A case of early-stage gastric cancer (well-differentiated adenocarcinoma, 0–IIc, 12 mm, blue arrowhead) located in the greater curvature of the lower body of the stomach with intestinal metaplasia and map-like redness (yellow arrow). A. map-like redness and early-stage gastric cancer using white-light imaging, B. map-like redness and gastric cancer using texture and colour enhancement imaging (TXI) mode 1, C. early-stage gastric cancer using narrow-band imaging and D. early-stage gastric cancer using TXI mode 1.

The Kyoto classification of gastritis also recommends using IEE to evaluate intestinal metaplasia [56,59,60]. However, it is unclear whether TXI modes 1 and 2 are useful modalities for assessing atrophy and intestinal metaplasia at health check-ups. Since gastric atrophy, intestinal metaplasia and map-like redness are observed through colour changes, TXI may improve their detection rates. To date, two studies have investigated the usefulness of TXI for detecting colour differences in gastric atrophy [7,15], intestinal metaplasia [7] and map-like redness [7], and the endoscopic visibility of gastric atrophy [15] (Tables 1 and 2, Figure 4). Ishikawa et al. [15] reported that atrophy is more visible in TXI mode 1 and mode 2 than in WLI, and that TXI mode 1 reveals a significantly greater colour difference in the tissue surrounding atrophy than WLI (colour differences: 14.2 ± 8.0 *vs.* 8.7 ± 4.2, *p* < 0.01). In our study, we found that although NBI and TXI mode 2 demonstrated comparable colour differences in the tissue surrounding atrophy, intestinal metaplasia and map-like redness, these techniques produced significantly greater colour differences in the tissue surrounding atrophy and intestinal metaplasia compared to WLI [7]. Therefore, using TXI mode 1, mode 2 or NBI for endoscopic screening at health check-ups may be more sensitive for visualizing atrophy and intestinal metaplasia by enhancing colour differences over WLI. Because TXI mode 1 especially enhances colour changes and improves visibility, this modality may be useful for detecting reddish-coloured intestinal metaplasia representative of map-like redness. Further, as atrophy and intestinal metaplasia are major risk factors for gastric cancer, large-scale multi-centre prospective studies are needed to investigate the efficacy of TXI modes 1 and 2 for detecting atrophy, intestinal metaplasia, and gastric cancer and for identifying those at high risk among not only *H. pylori*-eradicated patients but also *H. pylori*-positive patients.

**Table 2.** Visibility scores of oesophageal, gastric and duodenal diseases using various image-enhanced endoscopy techniques.

Disease		Number of patients /lesions	Factor	WLI	TXI mode 1	TXI mode 2	NBI	*p* Value (WLI *vs.* TXI mode 1)	*p* Value (WLI *vs.* TXI mode 2)	*p* Value (TXI mode1 * vs.* 2)
Oesophagus										
SCC	Dobashi et al. [14]^d^	30	Expert	–	0.68 ± 0.34	0.49 ± 0.37	0.91 ± 0.17	NA	NA	NA
		30	Non-expert	–	0.58 ± 0.33	0.31 ± 0.35	0.75 ± 0.31	NA	NA	NA
Stomach										
Atrophy	Ishikawa et al. [15]^a^	19/	All	2.8 ± 0.9	3.8 ± 0.5	3.5 ± 0.7	–	<0.01	<0.01	<0.01
			Expert	2.9 ± 0.8	3.8 ± 0.5	3.5 ± 0.7		<0.01	<0.01	<0.01
		–	Non-expert	2.7 ± 0.9	3.7 ± 0.6	3.5 ± 0.8	–	<0.01	<0.01	<0.01
Gastric cancer	Ishikawa et al. [15]^a^	11/12	All	2.0 ± 0.9	2.8 ± 1.0	2.2 ± 0.9	–	<0.01	<0.01	<0.01
		–	Expert endoscopist	2.0 ± 0.8	2.9 ± 0.8	2.3 ± 0.9	–	<0.01	<0.01	<0.01
		–	Trainee endoscopist	2.0 ± 0.9	2.6 ± 1.1	2.3 ± 1.0	–	<0.01	0.022	<0.01
		–	Indigo carmine	2.4 ± 1.1	2.7 ± 1.1	2.6 ± 1.1	–	<0.01	<0.01	0.070
		–	Indigo carmine: Expert	2.4 ± 1.1	2.9 ± 1.0	2.7 ± 1.1	–	<0.01	<0.01	0.050
		–	Indigo carmine: Trainee	2.4 ± 1.1	2.6 ± 1.1	2.5 ± 1.1	–	0.050	0.166	0.627
	Abe et al. (2022) [16]^b^	18/20	All	–	3.4 ± 2.0	3.0 ± 1.9	–	NA	NA	NA
Duodenum										
SNADET	Okimoto et al. [20]^c^	11/12	Surface structure: ICME	0.22 ± 0.87	1.58 ± 0.60	–	–	<0.001	NA	NA
		–	Vessel pattern: ICME	−1.19 ± 0.71	−0.58 ± 0.55	–	–	0.003	NA	NA

^a^Visibility score: 4, excellent (easily detectable); 3, good (detectable with careful observation); 2, fair (hardly detectable without careful examination); and 1, poor (not detectable without repeated careful examination).

^b^Visibility score: 2 (improved visibility), 1 (somewhat improved visibility), 0 (visibility equivalent to that of WLI), −1 (somewhat decreased visibility) and −2 (decreased visibility). Improved visibility was defined as a total score of 5 or more, no change as a score between −4 and 4, and decreased visibility as −5 or less.

^c^Visibility score: 2 (markedly improved visibility), 1 (improved visibility), 0 (unchanged visibility), − 1 (worsened visibility) and − 2 (markedly worsened visibility). Scores of 2 and 1 were defined as improved visibility.

^d^Visibility score: 1 (improved visibility of lesion), 0 (unchanged visibility of lesion) and −1 (worsened visibility of lesion).

ICME: magnified endoscopy with indigo carmine; NA: not available; NBI: narrow-band imaging; SCC: squamous cell carcinoma; SNADET: superficial nonampullary duodenal tumour; TXI: texture and colour enhancement imaging; WLI: white-light imaging

### 3.4. Gastric cancer

In 1994, the World Health Organization declared infection with *H. pylori* as an important risk factor for gastric cancer development [65]. Patients infected with *H. pylori* develop gastric cancer at a rate of 0.4% annually [57]. A meta-analysis reported that eradication therapy for *H. pylori* decreases the relative risk of gastric cancer to around 0.5 [66–68]. National gastric cancer screening programmes using endoscopy in Korea and Japan have contributed to a decrease in mortality by reducing the number of patients diagnosed at an advanced stage and through *H. pylori* eradication [1]. Although approximately 50,000 gastric cancer deaths have occurred annually in Japan over the past 40 years, deaths have significantly decreased in recent decades, from 50,136 in 2010 to 42,931 in 2019 [69,70].

Compared to a 5-year overall survival rate of >90% in patients with stage IA gastric cancer, survival in stage IV patients is <20%. Therefore, it is important to accurately detect gastric cancer as early as possible. WLI is the most common method for imaging the stomach and is known to detect lesions that show clear differences in colour shading or mucosal surface irregularities. However, WLI has difficulty recognizing lesions with little mucosal unevenness or only a slight change in colour tone. NBI can facilitate the diagnosis and identification of gastric cancer by illuminating mucosal surface structures at two narrow wavelengths [390–445 nm (blue light) and 530–550 nm (green light)] that are easily absorbed by blood haemoglobin and highlight the capillaries in the mucosal surface layer. However, a Japanese open-label RCT reported that the sensitivity of second-generation NBI (77.6%) for detecting gastric cancer in high-risk patients with a history of endoscopic resection for cancer was similar to that of WLI (72.5%), suggesting that second-generation NBI does not increase the detection rate over conventional WLI [71]. Consequently, the Japanese Guidelines for endoscopic diagnosis of gastric cancer state that the usefulness of IEE for the detection of early gastric cancer is under discussion (Evaluation by the modified Delphi method: 8, Strength of recommendation: None, Level of evidence: D) [72]. However, in comparison between LCI and WLI for detecting gastric cancer in a RCT, the percentage of patients with gastric tumours diagnosed in the first endoscopic examination was higher with LCI than with WLI [5.5% (41/750) *vs.* 3.3% (25/752), *p* = 0.011) and the proportion with overlooked neoplasms was lower in the LCI group than in the WLI group [5], suggested that LCI may be more effective than WLI for detecting gastric cancer [5,73,74]. Also, in a meta-analysis using reports investigated the usefulness of IEE (mostly NBI), the sensitivity and specificity for the diagnosis of intestinal metaplasia were 86 and 77%, and for dysplasia/early gastric cancer these values were 90 and 83%, respectively, and showed the usefulness of IEE for detection of gastric cancer [75]. In addition, according to the MAPS II guideline, an official statement from the European Society of Gastrointestinal Endoscopy, states that high-definition endoscopy with IEE is better than high-definition WLI alone for the diagnosis of gastric atrophy, intestinal metaplasia and gastric cancer [61]. Although LCI and NBI may have the potential to detect gastric cancer at high sensitivity and specificity, we think that continued efforts are needed to develop new IEE methods and to identify the most optimum strategy for detecting gastric cancer in clinical practice.

Clinical guidelines for gastric cancer do not currently mention using TXI for the detection and diagnosis of gastric cancer. Three reports to date have examined the usefulness of TXI for diagnosing gastric cancer, all of which studied a small number of target gastric cancer cases (Tables 1 and 2) [15,16]. In one study of 12 gastric cancer patients, the colour difference based on the CIE L*a*b* colour space system surrounding gastric cancer borders was significantly greater in TXI mode 1 than in WLI or TXI mode 2 [18.7 ± 16.0 (TXI mode 1) *vs.* 8.0 ± 4.2 (WLI) or 10.2 ± 8.4 (TXI mode 2)] using the GIF-EZ1500 and GIF290Z oral endoscopes [15]. However, TXI mode 2 showed no significant advantage in terms of colour difference over WLI (*p* = 0.831). Abe et al. [16] reported that, in 20 patients, the colour differences surrounding gastric cancer in WLI, TXI mode 1, and mode 2 using the GIF290Z oral endoscope were 10.3 ± 4.7, 15.5 ± 7.8 and 12.7 ± 6.1, respectively. Therefore, although TXI mode 1 was more effective for revealing colour differences in the tissue surrounding gastric cancer than WLI, there was no significant difference between TXI modes 1 and 2. Greater contrast in colour tone can be obtained by combining TXI, especially TXI mode 1, with WLI, which may contribute to improving the identification rate of early-stage gastric cancer in endoscopic screening. Since the image in TXI mode 1 is colour-enhanced, TXI mode 1 may have an advantage over mode 2 for those examining colour differences based on the CIE L*a*b* colour space system. TXI mode 1 may reveal gastric cancer, especially reddish-coloured cancer, through enabling greater contrast than WLI or TXI mode 2.

In terms of the visibility of gastric cancer using WLI and TXI, Ishikawa et al. [15] compared images taken by six endoscopists and showed that gastric cancer was significantly more visible in TXI mode 1 and TXI mode 2 than in WLI. Abe et al. [16] reported that visibility improves in 35% and 20% of cases in TXI mode 1 and TXI mode 2, respectively, compared to WLI, especially in patients with macroscopic type 0–IIc or 0–IIb [visibility score: 2 (markedly improved), 1 (improved), 0 (unchanged), −1 (worsened), −2 (markedly worsened). Scores of 2 and 1 were defined as improved visibility]. The visibility of gastric cancer has no association with atrophy, *H. pylori* infection status, tumour histology, location, size or depth [16].

A case reports have also reported the efficacy of TXI for detecting early gastric cancer, such as combining TXI mode 1 with indigo carmine dye spraying to highlight the border of slightly depressed lesions [76].

Although both TXI modes 1 and 2 are more advantageous for detecting and visualizing gastric cancer than WLI alone, TXI mode 1 produces the greatest colour differences and visibility scores, suggesting that this mode may be the best option for detecting gastric cancer at health check-ups. In addition, because post-eradication gastric cancer appears as characteristic small and reddish shallow depressions, TXI mode 1 may be useful for improving the visibility of gastric cancer in patients who have received eradication therapy. In the future, it will be important to compare TXI modes 1 and 2 with NBI for detecting gastric cancer and to evaluate the types of gastric cancer for which TXI is useful or weak.

### 3.5. Superficial nonampullary duodenal tumour

Detection of superficial non-ampapullary duodenal epithelial tumours (SNADETs) has increased recently at endoscopic screenings, with a reported detection rate of around 1.5–4.6% [77]. Because duodenal adenocarcinoma has the lowest 5-year survival rate of all the small intestinal carcinomas (less than 30% [78], reliable endoscopic detection in the early stages and selection of appropriate treatment are particularly important [42,79,80]. The diagnostic algorithm for SNADETs that uses magnifying endoscopy with NBI for visualizing microsurface structures and microvessel patterns may be useful for differentiating between low-grade dysplasia (LGD) and high-grade dysplasia (HGD) or adenocarcinomas [81]. The European Society of Gastrointestinal Endoscopy Guideline suggests the use of magnifying chromoendoscopy for endoscopic diagnosis and staging of SNADETs [82]. In a Japanese multicentre trial, 31% of SNADET patients were diagnosed with LGD, 28% with HGD, and 41% with duodenal cancer [42]. In terms of the endoscopic and pathological features of SNADETs, magnifying endoscopy with NBI shows that white opaque substance (22.2% in gastric phenotype *vs.* 89.7% in intestinal phenotype, *p* < 0.01) and light blue crest (0 *vs.* 43.6%, *p* < 0.05) are significantly less frequently observed in the gastric phenotype than the mucin phenotype [83]. An important consideration for SNADETs in clinical practice is that fibrosis caused by the biopsy procedure may hinder endoscopic treatment. Further, as recent studies have reported limited diagnostic performance for endoscopic duodenal biopsy sampling, pathological evaluation of SNADETs requires more caution than that for gastric cancer and oesophageal cancer [82].

**Figure 5.**
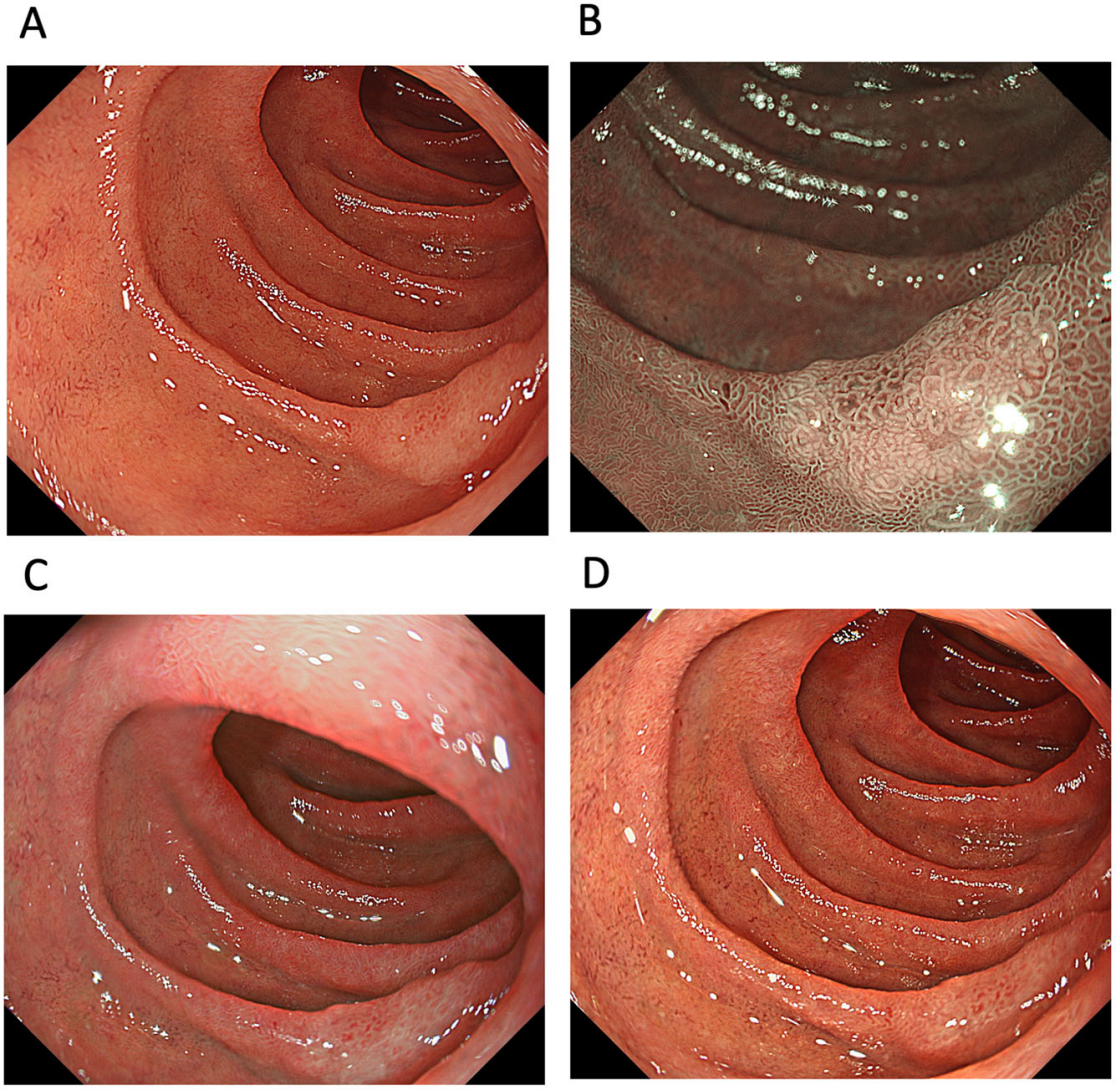
A case of superficial nonampullary duodenal tumour. The tumour observed using (A) white-light imaging, (B) narrow-band imaging, (C) texture and colour enhancement imaging (TXI) mode 1 and (D) TXI mode 2.

The macroscopic presentation for sporadic duodenal adenoma is mainly milk-white or reddish mucosa. European guidelines do not mention using TXI for detecting and diagnosing SNADET [82]. In a pilot study in 12 patients with SNADETs, Okimoto et al. [20] investigated the usefulness of TXI for diagnosing SNADETs. Compared with the visibility score obtained using magnified endoscopy with NBI, scores for the surface structure of SNADETs were 0.08 ± 0.81 using magnified endoscopy with TXI, 0.22 ± 0.87 using magnified endoscopy with indigo carmine (ICME)-WLI and 1.58 ± 0.60 with ICME-TXI [visibility score: 2 (markedly improved), 1 (improved), 0 (unchanged), −1 (worsened), −2 (markedly worsened)] (Table 2) [20]. Importantly, these results indicate that the visibility of the surface structure does not improve with indigo carmine or texture enhancement by TXI alone. Therefore, ICME-TXI facilitates the visibility of the surface structure of SNADETs and may facilitate preoperative diagnosis of SNADETs (Figure 5).

### 3.6. Colorectal tumours: adenoma and cancer

Colorectal cancer is a common malignancy worldwide, especially in Western countries, and the number of patients diagnosed with and dying of colorectal is rising [84]. Although older age, male sex, family history of colorectal cancer, obesity and red meat intake are major risk factors of colorectal adenoma and cancer, endoscopic screening and surveillance for colorectal cancer and resection of colorectal adenoma can reduce disease mortality [85]. In fact, the Japanese guideline for colonoscopy screening and surveillance recommends endoscopic resection of neoplastic lesions including colorectal polyps and cancers to reduce colorectal cancer mortality [84]. Evidence shows that endoscopic resection by total colonoscopy reduces cancer incidence by 43–90% [86] and cancer mortality by 53–88% [85,87]. Guidelines in Western countries recommend performing endoscopic resection after a surveillance period of 5–10 years for low-risk patients and 3 years for high-risk patients [84,88,89].

**Figure 6.**
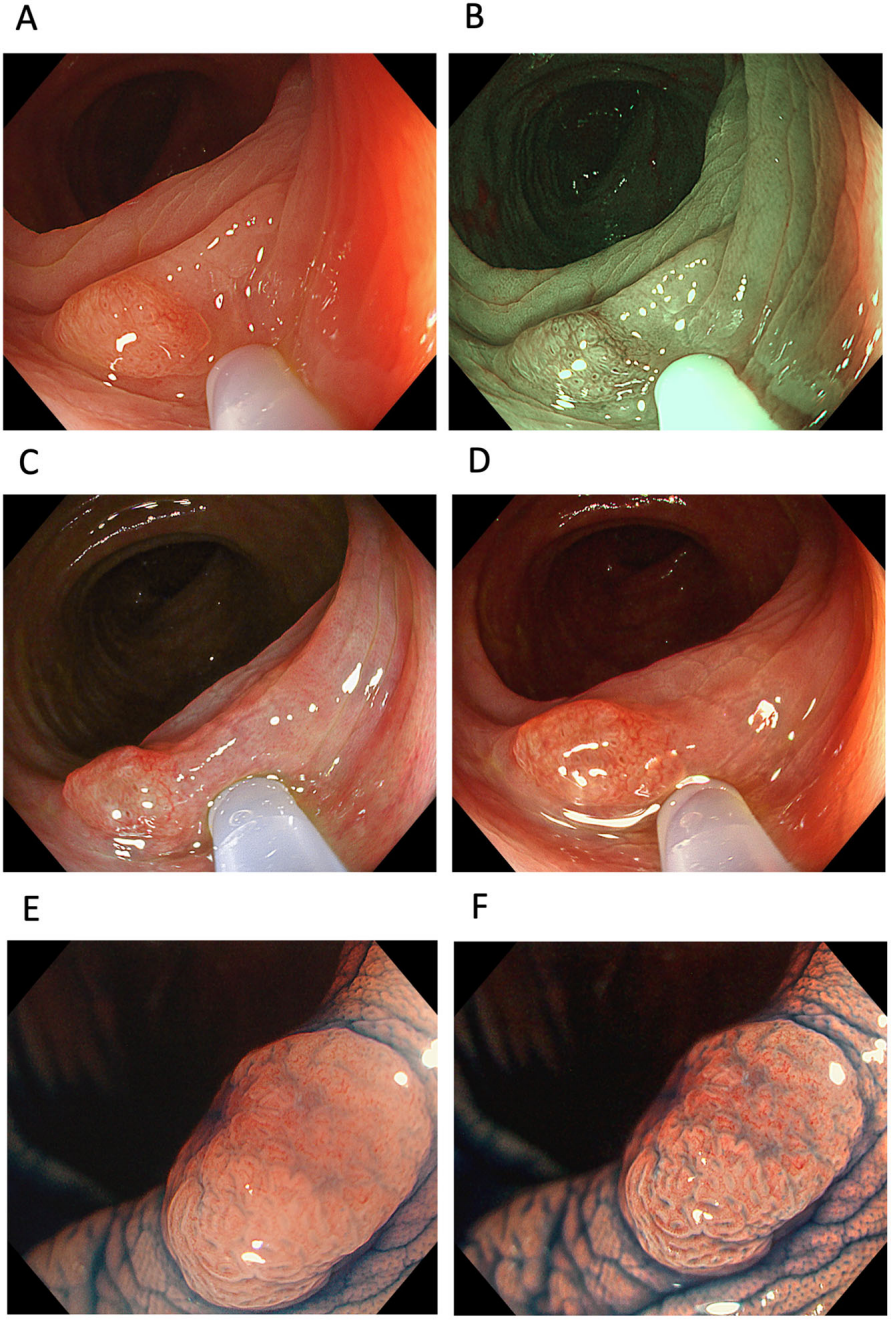
Cases of colon polyp. A tumour in one case observed using (A) white-light imaging (WLI), (B) narrow-band imaging, (C) texture and colour enhancement imaging (TXI) mode 1 and (D) TXI mode 2. A polyp observed in another case using (E) magnified endoscopy with indigo carmine (ICME)-WLI and (F) ICME-TXI mode 1.

The miss rate for colorectal polyps using WLI alone is 22–28% [90]. The Japanese guideline states that optical digital methods including conventional NBI and LCI have comparable efficacy for detecting colorectal lesions to WLI [84,91,92]. However, a recent meta-analysis showed that NBI detects colorectal adenoma at a higher rate than WLI [93]. Also in meta-analysis using RCTs and prospective studies comparing LCI with WLI for detection of colorectal adenoma detection, LCI showed significant superiority for adenoma detection compared with WLI (RR: 1.26, 95% CI 1.14–1.39 *p* < 0.001 for adenoma detection) [94] and this efficacy of LCI is shown in not only Japan but also Western countries [94–96]. A recent RCT showed that NBI has a significant advantage over LCI for detecting colorectal polyps (71.3 *vs.* 55.9%; *p* = 0.008), serrated lesions (34.6 *vs*. 22.1%; *p* = 0.02), and mean the number of polyps (2.04 *vs.* 1.35; *p* = 0.02) [97]. However, no study has examined the efficacy of TXI for detecting and diagnosing colorectal polyps and cancer (Tables 1 and 3, and Figure 6) and the development of a technique that can increase the detection rate of adenoma in all patients is still required. In a preliminary study of 101 colorectal lesions, Yoshida et al. [19] reported that while the colour difference of non-polypoid lesions was higher in TXI mode 1 (13.3 ± 6.3) than WLI (9.7 ± 6.0, *p* < 0.001), it was similar to that in NBI (13.1 ± 6.8). Further, the colour difference observed for LGD + HGD + T1 lesions in TXI mode 1 was significantly higher than that in WLI [19]. However, despite the objective colour difference in TXI mode 1 being greater than that in WLI and comparable to that in NBI, the detection efficacy based on colour differences in TXI mode 1 can differ in different pathological diseases.

**Table 3.** Visibility scores of colonic diseases using various image-enhanced endoscopy techniques.

Disease		Number of patients /lesions	Factor	WLI	TXI mode 1	TXI mode 2	NBI	*p* Value (WLI *vs.* TXI mode 1)	*p* Value (WLI *vs.* TXI mode 2)	*p* Value (NBI *vs.* TXI mode 1)	*p* Value (TXI mode 1 *vs.* 2)
Colorectal adenoma	Toyoshima et al. [21]^a^	37/61	Tumour margin	2.86 ± 1.02	3.47 ± 0.79	–	3.76 ± 0.52	<0.001	NA	<0.001	NA
		–	Vessel pattern	2.17. ± 0.90	3.05 ± 0.79	–	3.79 ± 0.47	<0.001	NA	<0.001	NA
		–	Surface pattern	1.95 ± 0.79	2.89 ± 0.85	–	3.67 ± 0.55	<0.001	NA	<0.001	NA
Colorectal lesion (cancer, adenoma and SSL)	Tamai et al. [22]^b^	22/68	Total	70.0 ± 20.1	80.5 ± 18.6	75.6 ± 18.1	69.0 ± 20.6	<0.01	NA	<0.01	<0.05
		–	Flat lesion	64.1 ± 21.2	76.5 ± 20.2	71.8 ± 19.4	64.2 ± 22.0	<0.01	NA	<0.01	0.02
		–	Non-flat lesion	78.8 ± 14.6	86.6 ± 14.0	81.3 ± 14.3	76.0 ± 15.9	<0.01	NA	<0.01	<0.05
Serrated polyps	Nishizawa et al. [23]^a^	27/29	All serrated polyps	2.27 ± 0.75	2.93 ± 0.76	–	2.74 ± 0.79	<0.001	NA	NS	NA
		/18	SSLs	2.25 ± 0.76	2.90 ± 0.77	–	2.65 ± 0.79	<0.001	NA	NS	NA
		/11	HP	2.30 ± 0.72	2.97 ± 0.67	–	2.88 ± 0.77	<0.001	NA	NS	NA
		–	All: vessel pattern	2.30 ± 0.74	2.91 ± 0.80	–	3.23 ± 0.84	<0.001	NA	NS	NA
		–	All: surface pattern	1.86 ± 0.64	2.75 ± 0.68	–	3.46 ± 0.70	<0.001	NA	<0.001	NA
		–	SSL: vessel pattern	2.24 ± 0.74	2.89 ± 0.81	–	3.19 ± 0.86	<0.001	NA	NS	NA
		–	SSL: surface pattern	1.80 ± 0.62	2.70 ± 0.66	–	3.39 ± 0.78	<0.001	NA	<0.001	NA
		–	HP: vessel pattern	2.41 ± 0.73	2.96 ± 0.79	–	3.33 ± 0.77	NS	NA	NS	NA
		–	All: surface pattern	2.00 ± 0.67	2.85 ± 0.70	–	3.59 ± 0.49	<0.01	NA	<0.01	NA
Non-polypoid colorectal lesions (<20 mm)	Yoshida et al. [19]^a^	26/101	–	2.85 (2.49, 3.20)	3.42 (3.06, 3.77)	–	3.33 (2.98, 3.69)	<0.01	NA	0.258	NA
		–	Trainee endoscopist	2.84 (2.38,3.31)	3.38 (2.91, 3.85)	–	3.29 (2.82, 3.76)	<0.01	NA	0.445	NA
		–	Expert endoscopist	2.85 (2.45, 3.24)	3.45 (3.06, 3.85)	–	3.37 (2.98, 3.77)	<0.01	NA	0.388	NA
		–	Right-side	2.73 (2.30,3.16)	3.33 (2.89, 3.76)	–	3.15 (2.71, 3.58)	<0.01	NA	0.068	NA
		–	Left-side	3 (2.52, 3.48)	3.54 (3.06, 4.01)	–	3.58 (3.10, 4.06)	<0.01	NA	0.689	NA
		–	≥10 mm	3.11 (2.53, 3.69)	3.62 (3.04, 4.20)	–	3.49 (2.91, 4.07)	<0.01	NA	0.336	NA
		–	<10 mm	2.65 (2.29, 3.01)	3.26 (2.90, 3.62)	–	3.22 (2.86, 3.57)	<0.01	NA	0.548	NA
		–	SSL + HP	2.78 (2.32, 3.24)	3.38 (2.92, 3.85)	–	3.29 (2.83, 3.75)	<0.01	NA	0.398	NA
		–	LGD + HGD + T1	2.93 (2.51, 3.36)	3.46 (3.03, 3.88)	–	3.38 (2.96, 3.81)	<0.01	NA	0.44	NA

^a^Visibility score: 4, excellent (easily detectable); 3, good (detectable with careful observation); 2, fair (hardly detectable without careful examination); and 1, poor (not detectable without repeated careful examination).

^b^Visual analogue scale: 0, worst; 25, poor; 50, acceptable; 75, good; and 100, best visualization of the lesion.

HGD: high-grade dysplasia; HP: hyperplastic polyp; LGD: low-grade dysplasia; NA: not available; NBI: narrow-band imaging; NS: not significant; SSL: sessile serrated lesions; TXI: texture and colour enhancement imaging; WLI: white-light imaging

Second-generation NBI improves the visibility of colorectal polyps compared to WLI, irrespective of colorectal polyp characteristics, such as location, size, histopathology, and morphology and endoscopist experience [98]. In a study from the same hospital as that of the Yoshida et al. study, the visibility score of TXI mode 1 (3.42 [95% CI: 3.06–3.77]) for colorectal polyps was significantly higher than that of WLI (2.85 [2.49–3.20], *p* < 0.001) but not that of NBI (3.33 [2.98–3.69], *p* = 0.258) [visibility score: 4, excellent; 3, good (detectable with careful observation); 2, fair (hardly detectable without careful examination); and 1, poor], irrespective of endoscopist experience or size (10 mm), location (right-side and left-side), and histopathology (sessile serrated lesions, hyperplastic polyp, dysplasia and cancer) of the polyp (Table 3) [19]. Nishizawa et al. [23] focused on the association between serrated polyps and TXI mode 1, and showed that TXI mode 1 produced significantly higher visibility scores for detection, vessel pattern, and surface pattern than WLI for sessile serrated lesions and hyperplastic polyps. However, visibility of serrated colorectal polyps in TXI mode 1 was lower than that using chromoendoscopy. Further, the team showed that magnified TXI was inferior to magnified NBI for observing surface patterns [23]. Moreover, Tamai et al. [22] examined video clips of lesions taken using WLI, TXI mode 1, TXI mode 2, and NBI, and reported mean visualization scores of 70.0 ± 20.1, 80.5 ± 18.6, 75.6 ± 18.1 and 69.0 ± 20.6, respectively, where 0 indicates worst, 25 indicates poor, 50 indicates acceptable, 75 indicates good and 100 indicates the best visualization of the lesion. They concluded that TXI enables better visualization of colorectal lesions, even flat lesions, than WLI and NBI.

A small number of case reports have also reported the efficacy of TXI for detecting diseases, such as combining TXI mode 1 with indigo carmine dye spraying to highlight the border of flat ulcerative colitis-associated neoplasia [99]. Although further large-scale studies are needed to determine the efficacy of TXI for detecting colorectal polyp cancer, TXI may be a promising technology for improving the detectability of colorectal lesions (Figure 6).

## 4. Summary

The benefits of TXI in clinical practice include the ability to obtain images in a WLI-like tone compared with NBI and BLI, and to detect gastrointestinal diseases, such as ESCC, EAC, gastric cancer, intestinal metaplasia and colorectal polyp, based on colour differences. TXI, a novel IEE, brightens dark areas in WLI images, enhances textures, including subtle surface elevations or depressions, and enhances colour differences between the surrounding mucosa of gastrointestinal lesions. In particular, the enhancement of texture and brightness with TXI mode 2 enables the detection of gastrointestinal diseases using existing diagnostics, and is ideal for use in the first screening observation of the gastrointestinal tract. Further, the enhancement of texture, brightness and colour observed with TXI mode 1 makes it a promising modality for visualizing morphological and colour differences in gastrointestinal diseases compared with WLI. TXI mode 1 can therefore improve the visibility of gastrointestinal diseases and qualitative diagnosis, especially for diseases associated with colour changes, such as red-coloured cancers, map-like redness, reflux esophagitis, inflammation-related diseases, whitish-coloured cancers (diffuse-type adenocarcinoma) and MALT lymphoma [13]. However, all reports of the usefulness of TXI to date have examined a small number of cases and the data available at screening endoscopy in daily clinical practice is still preliminary. The clinical usefulness of TXI mode 1 and mode 2 for detecting and evaluating gastrointestinal diseases at endoscopic screening should therefore be confirmed in a large-scale prospective multicentre study that preferably compares the findings with those from other IEEs, such as NBI, BLI and LCI.

## Data Availability

The data based on the results of this study were obtained, are accessible from the corresponding authors upon reasonable request.
